# Machine learning‐based classifying of risk‐takers and risk‐aversive individuals using resting‐state EEG data: A pilot feasibility study

**DOI:** 10.1002/brb3.3139

**Published:** 2023-06-27

**Authors:** Reza Eyvazpour, Farhad Farkhondeh Tale Navi, Elmira Shakeri, Behzad Nikzad, Soomaayeh Heysieattalab

**Affiliations:** ^1^ Department of Biomedical Engineering, School of Electrical Engineering Iran University of Science and Technology (IUST) Tehran Iran; ^2^ Department of Cognitive Neuroscience University of Tabriz Tabriz Iran; ^3^ Department of Business Management, Faculty of Management and Accounting Allameh Tabataba'i University Tehran Iran; ^4^ Neurobioscince Division Research Center of Bioscience and Biotechnology, University of Tabriz Tabriz Iran

**Keywords:** decision‐making, machine learning, manager, resting‐state EEG, support vector machine classifier

## Abstract

**Background:**

Decision‐making is vital in interpersonal interactions and a country's economic and political conditions. People, especially managers, have to make decisions in different risky situations. There has been a growing interest in identifying managers’ personality traits (i.e., risk‐taking or risk‐averse) in recent years. Although there are findings of signal decision‐making and brain activity, the implementation of an intelligent brain‐based technique to predict risk‐averse and risk‐taking managers is still in doubt.

**Methods:**

This study proposes an electroencephalogram (EEG)‐based intelligent system to distinguish risk‐taking managers from risk‐averse ones by recording the EEG signals from 30 managers. In particular, wavelet transform, a time‐frequency domain analysis method, was used on resting‐state EEG data to extract statistical features. Then, a two‐step statistical wrapper algorithm was used to select the appropriate features. The support vector machine classifier, a supervised learning method, was used to classify two groups of managers using chosen features.

**Results:**

Intersubject predictive performance could classify two groups of managers with 74.42% accuracy, 76.16% sensitivity, 72.32% specificity, and 75% *F*1‐measure, indicating that machine learning (ML) models can distinguish between risk‐taking and risk‐averse managers using the features extracted from the alpha frequency band in 10 s analysis window size.

**Conclusions:**

The findings of this study demonstrate the potential of using intelligent (ML‐based) systems in distinguish between risk‐taking and risk‐averse managers using biological signals.

## Graphical abstract

Decision‐making is vital in interpersonal interactions and a country's economic and political conditions. People, especially managers, have to make decisions in different risky situations. There has been a growing interest in identifying managers' personality traits (i.e., risk‐taking or risk‐averse) in recent years. Although there are findings of signal decision‐making and brain activity, the use of an intelligent brain signal‐based system to predict risk‐averse and risk‐taking managers is still unknown. This study proposes an electroencephalogram (EEG)‐based intelligent system to distinguish risk‐taking managers from risk‐averse ones by recording the EEG signals from 30 managers. In particular, wavelet transform, a time‐frequency domain analysis method, was used on resting‐state EEG data to extract statistical features. Then, a two‐step statistical wrapper algorithm was used to select the appropriate features. The support vector machine (SVM), a supervised learning method, was used to classify two groups of managers using chosen features. Inter‐subject predictive performance could classify two groups of managers with 72.10% accuracy, 74.41% sensitivity, 69.79% specificity, and 72.31% F1‐Measure, indicating that machine learning (ML) models can distinguish between risk‐taking and risk‐averse managers using the features extracted from the Gamma band. The findings of this study demonstrate the high potential of using intelligent systems in selecting managers using biological signals.

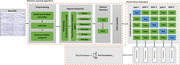

## 1 INTRODUCTION

Decision‐making is a complex cognitive process and an essential skill in everyday life (Poudel et al., 2020; Si et al., 2020; Wojcik et al., 2019). It becomes imperative when individuals manage an organization or country, and the outcome of the decision can be decisive in economic, cultural, and political contexts. Decision‐making has been part of studies of neuroscience, neuroeconomics, and related disciplines for many decades (Krain et al., 2006; Opris et al., 2020; Srivastava et al., 2020; Viacava et al., 2016; Zhang, 2018). Understanding the neural and cognitive foundations of the decision‐making process is an important issue, especially in decisions made by managers. This information can play a vital role in selecting managers for an organization or even a country. One of the reasons that different decisions are made under the same conditions is heterogeneity in brain physiology. In this regard, the functional characteristics of the brain have been evaluated by examining the brain's activity at the resting‐state (Studer et al., 2013). The resting‐state functional connectivity, representing intrinsic brain activity, has been used to examine individuals’ impulsive economic decision‐making (Li et al., 2013). Along with this idea, electroencephalography (EEG) is a powerful non‐invasive tool for investigating the brain bases of human psychological processes (Li et al., 2020). The method has been applied in various decision‐making research domains (Ivaskevych, 2019; Lee et al., 2017; Pornpattananangkul et al., 2019; Ramsøy et al., 2018; Si et al., 2020; Wilson & Vassileva, 2018; Zheng et al., 2020). The use of intelligent systems based on learning from EEG signals has also been considered by most researchers (Al‐Nafjan et al., 2017; Anjum et al., 2020; Ieracitano et al., 2020; Maitín et al., 2020; Noor & Ibrahim, 2020; Rasheed et al., 2020; Roy et al., 2019; Tzimourta et al., 2021). Hence, developing intelligent EEG‐based systems to evaluate human decision‐making skills based on brain signals has become a challenging and demanding research area. For instance, Si et al. (2020) proposed an EEG‐based machine learning (ML) model to predict individuals’ responses. The authors could achieve higher accuracy by extracting discriminative spatial network pattern (DSNP) features from single‐trial EEG data and the Linear Discriminate Analysis (LDA)‐based model. Using two EEG datasets, they obtained predictive performance in the first and the second sets with 88% and 90% accuracies, respectively, in individual response detection. In a similar attempt, Wojcik et al. (2019) used seven classifications, namely, logistic regression, decision jungle, support vector machine (SVM), boosted decision tree, averaged perceptron, Bayes point machine, classic neural network, and locally‐deep support vector, to identify and classify reward/punishment properties in cortical activity. The authors obtained the best result using the locally‐deep support vector classifier with an accuracy of 69.8%.

The proposed ML models (Si et al., 2020; Wojcik et al., 2019) focus only on classifying individual responses, which are ML models of an intra‐subject classification. Due to the importance of decision‐making by managers, the automatic cross‐subject separation can also divide managers into two groups, risk‐taking and risk‐averse, based on the features extracted from the EEG signal. This study proposes a model of ML to achieve this goal. Resting‐state EEG data were recorded from managers, and the ML model was trained using information extracted from the collected signal of managers. Finally, using brain signals, this trained model can separate risk‐taking and risk‐averse managers.

## 2 MATERIALS AND METHODS

### 2.1 Participants

In this study, we recruited participants from a population of 173 managers who are responsible for formulating their organization's strategies and goals, and regularly encounter unplanned decisions. As gender differences can also affect decision‐making (Lin et al., 2019; Mahaldar & Aditya, 2017; Mehta, 2020), all participants were selected from the same gender in this study. The participants completed a 13‐item risk‐tolerance scale questionnaire to assess their willingness to engage in risky financial behavior (Grable, 1999; Grable & Lytton, 2003). Based on the questionnaire scores, managers were categorized as risk‐takers (scores > 32) or risk‐averse (scores < 22) in the economic decision‐making process. In addition, they were asked to evaluate themselves as risk‐takers or risk‐averse. Among them, the thirty healthy men (*N* = 30; mean age = 40.80; age range = 32–55 years) were selected for EEG data acquisition in this study. After ensuring the use of no drug or alcohol by participants on the day of data collection, the experimental procedures were explained orally. All participants also approved consent forms. The experimental study was approved by the Ethics Committee of the University of Tabriz (Tabriz, Iran) and was adapted to the regulations of the Declaration of Helsinki.

### 2.2 EEG recording and experimental procedures

EEG data were recorded using an ANT Neuro system (DC‐Amplifier, ANT Neuro, the Netherlands) with 64 scalp Ag/AgCl electrodes (waveguard cap, ANT Neuro, the Netherlands) positioned according to the 10/20 system. Cortical data were acquired at 250 Hz, and the impedance of all electrodes was maintained below 5 kΩ during data recording. The electrode AFz was also considered the ground and with the reference at the right earlobe (A2). EEG signals were collected in a room with sufficient light. Participants were asked to sit quietly in a chair while collecting data and refrain from moving their heads frequently. EEG data were collected at resting state, whereas both eyes were closed for 5 min.

### 2.3 EEG preprocessing and analysis strategy

All data were processed in MATLAB R2014b. The EEG data were preprocessed using an EEGLAB open‐source toolbox (Delorme & Makeig, 2004), made freely available by the Schwartz Center for Computational Neuroscience. The Butterworth band‐pass filter was applied to the EEG signal to filter the signal in the 0.1–85 Hz frequency range to eliminate high‐frequency noise. The filter also removed the 50‐Hz noise caused by the power line. A common average reference filter was applied to control the problems related to the signal‐to‐noise ratio (Ludwig et al., 2009). In addition, the independent component analysis was used to manually separate and remove artifactual components from EEG signals.

**FIGURE 1:**
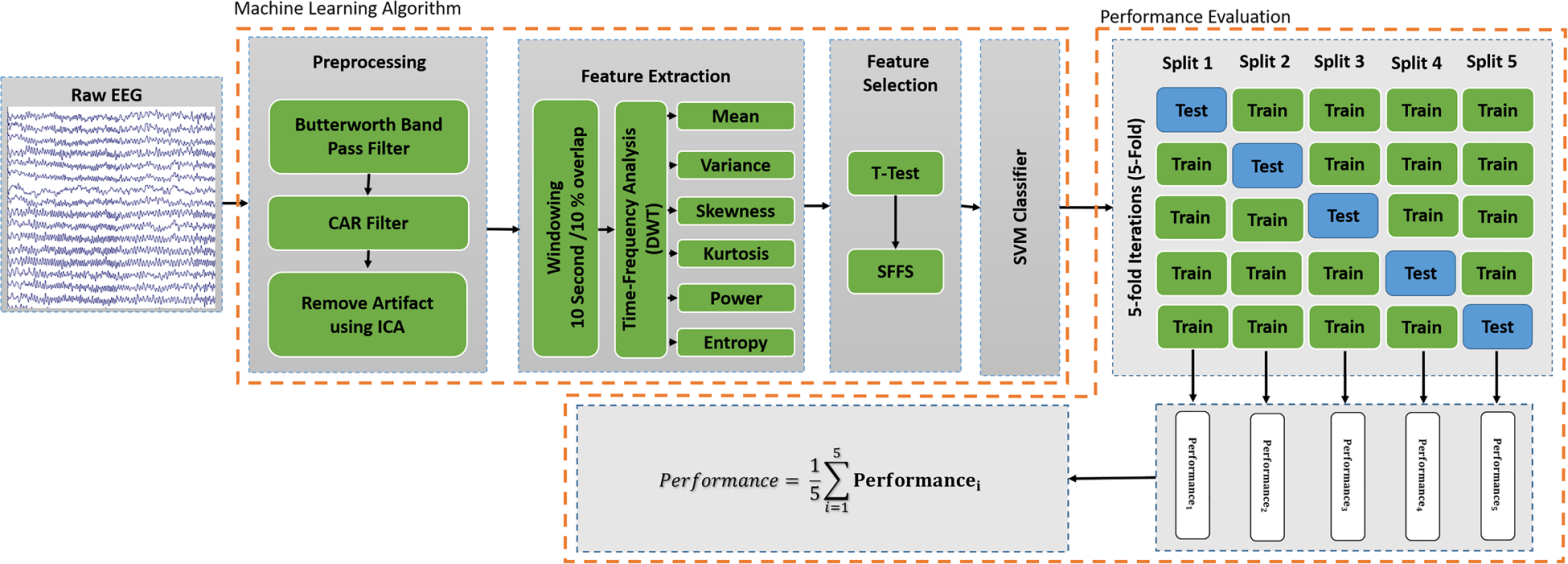
Flowchart of the electroencephalography (EEG) analysis strategy.

The current study used ML analysis to detect risk‐taking and risk‐averse managers. After the preprocessing of the brain signal, the feature extraction, feature selection, and SVM classifier are considered the consecutive steps of the ML algorithm (Figure 1). Raw EEG signals contain noise and redundant information that is not related to risk‐taking behavior. Therefore, to distinguish between the two groups, it is necessary to extract meaningful features from the signals. Feature extraction helps to reduce the amount of redundant data and allows the construction of a model with less machine effort, leading to faster learning and better generalization performance. By extracting relevant features, we can identify the essential characteristics of the EEG signals that differentiate risk‐taking and risk‐averse individuals. These features provide valuable insights into the underlying neural mechanisms of risk‐taking behavior and can be used to improve the accuracy of classification models. Frontal region EEG signals have been used in the time‐frequency domain for feature extraction (Bartra et al., 2013; Gianotti et al., 2009; Krain et al., 2006; Schutter & Van Honk, 2005; Si et al., 2019; Studer et al., 2013). The ML algorithm uses a two‐step process to select the appropriate feature, including statistical analysis to remove bad features and the sequential floating forward selection (SFFS) algorithm (Pudil et al., 1994) to select the feature perfectly. In the ML model, the SVM classifier is used to classify two groups of participants based on the extracted features of the EEG signal. This study uses the *K*‐fold cross‐validation algorithm to evaluate the ML model and the training and testing process. More details of each step of the ML algorithm are described below.

### 2.4 Machine learning analysis

#### 2.4.1 Feature extraction and selection

**FIGURE 2:**
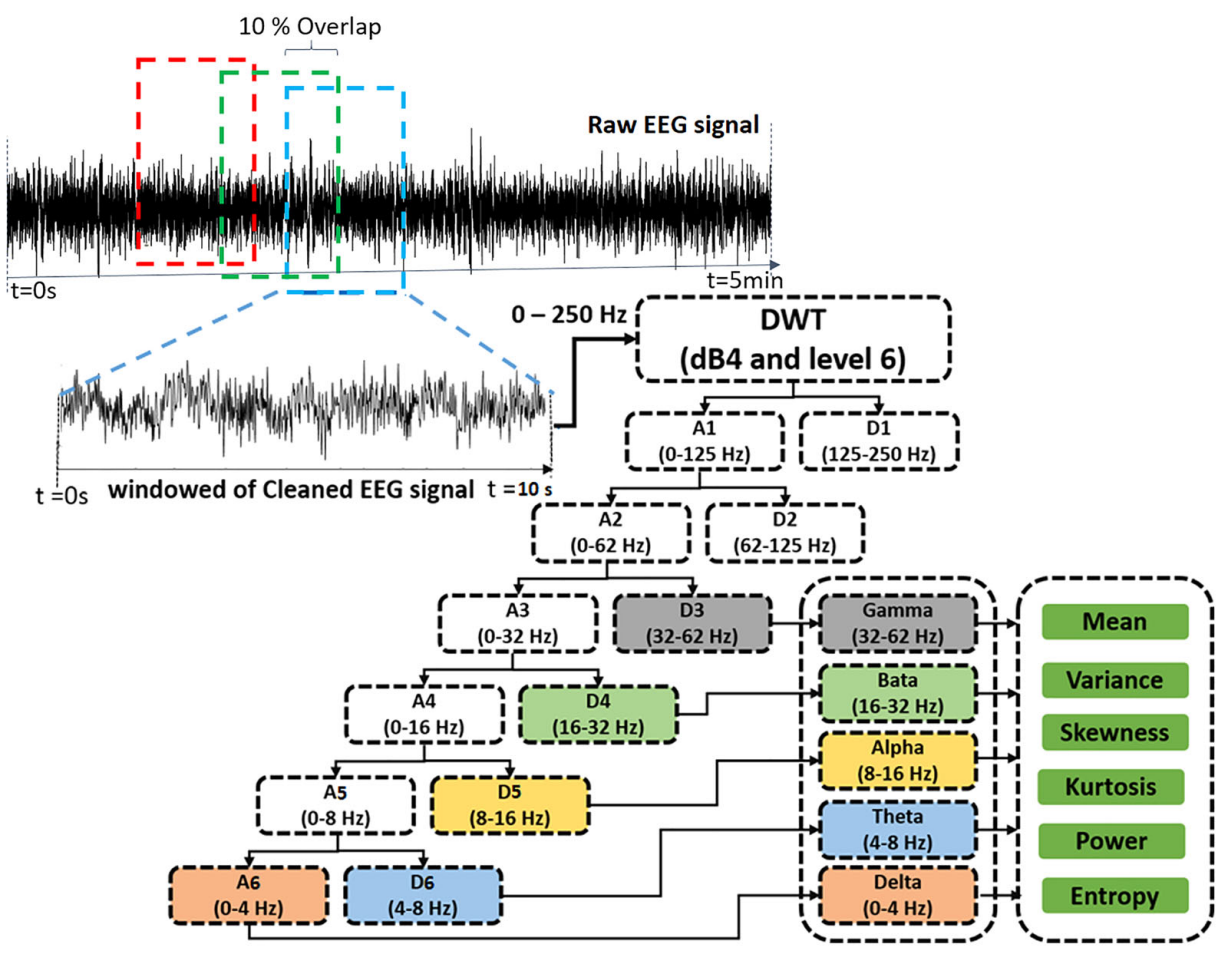
Extracting electroencephalography (EEG) features using discrete wavelet transform (DWT) with the mother wavelet dB4 and level 6.

The EEG data as a complex signal contains much information about brain neural patterns. The main purpose of extracting features from the brain signal is to extract meaningful information that can be used in the ML model to classify the two groups of managers. In the feature extraction step, cleaned 5‐min EEG signals in consecutive 10‐s time windows with 10% overlap have been transformed into feature vectors. Time‐frequency analysis was used to extract the features to overcome the nonstationary nature of the EEG signal (Bajaj, 2021; Ieracitano et al., 2020; Jacob et al., 2018). This study used the discrete wavelet transform (DWT) with the mother wavelet dB4 and level 6 as a time‐frequency analysis, as shown in Figure 2. In this work, the DWT was utilized to extricate the frequency sub‐bands of EEG signals by reorganizing the coefficients vector of type based on the wavelet decomposition structure. Utilizing the gotten to DWT‐based time‐frequency representation, the EEG signals of all frequency sub‐bands are extricated from the computed detailed and approximate coefficients. At that point, a set of features were extricated from each computed sub‐band EEG signals. Six features, including mean amplitude, variance, skewness, kurtosis, power, and Shannon entropy, were extracted from the frontal region with 16 channels and 5 frequency bands (delta [0.1–4 Hz], theta [4–8 Hz], alpha [8–16 Hz], beta [16–32 Hz], and gamma [32–62 Hz]). Previous studies have shown decision‐making in the brain's frontal and prefrontal areas (Ramsoy et al., 2018; Studer et al., 2013, Gianotti et al., 2009). Therefore, the frontal region (FP1, FP*z*, FP2, AF7, AF3, AF4, AF8, F7, F5, F3, F1, F*z*, F2, F4, F6, and F8) was considered in selecting the channels to extract their features. Therefore, for 5‐min EEG signal of every participant the feature vector containing 15,840 data (33 [10‐s time windows] × 6 [features] × 5 [frequency bands] × 16 [channels]).

After feature extraction, the feature selection step was performed to reduce the number of features and the computational cost and, in some cases, to improve the performance of the ML model. Selecting features among extracted features from high‐dimensional EEG data, eliminating irrelevant, redundant, or noisy features, and selecting the appropriate features help identify and diagnose brain conditions easily. Consequently, this facilitates distinguishing between the two groups of managers based on the cortical signal. As shown in Figure 1, the filter‐based and wrapper‐based techniques were used consecutively to select the features. Filter feature selection methods use statistical techniques to eliminate features that cannot distinguish between the two groups of managers outside of the predictive models. The *t*‐test was used to compare the extracted features between groups. Then, the features that did not show a significant difference between the two groups were removed from the feature vector.

In the wrapper method, adding and removing predictors selected in the previous step tries to achieve an optimal combination of features to maximize the model's performance. In this selection method, the algorithm is not concerned with the feature types and selects a subset of features that show the best‐performing model according to the performance metric. In this study, the Student *t*‐test was used to remove inappropriate features and the SFFS algorithm (Pudil et al., 1994) as the wrapper method that dynamically increases and decreases the number of features to find an appropriate combination of remaining features.

#### 2.4.2 Classification model

One of the most widespread algorithms that researchers have used for EEG classification is the SVM classifier algorithm. The SVM is a supervised ML model that can classify high‐dimensional feature space based on the hyperplane (Shanir et al., 2018; Wang et al., 2022). This classification algorithm reduces the time required for the learning phase by transforming the prediction problem into an optimization problem and has better accuracy and speed than the other algorithms. In this study, the SVM classification algorithm was used to classify participants into two groups, risk‐taking, and risk‐averse managers, based on the information learned from the features extracted from resting‐state EEG signals.

To evaluate the classification algorithm in a range of features, a fivefold cross‐validation method was adopted in the proposed study. This evaluation method has better validity due to the limited number of subjects. As shown in Figure 1, the subjects are divided into five equal portions using random selection in the fivefold validation method. The ML model is trained using four portions for testing with the remaining portion. This process is repeated five times, changing the test and training portion at each stage. In each repetition, the values of different performance parameters are obtained, and the average of the results is defined as the performance of the ML model. The classification results have been evaluated using four metrics: accuracy (%), sensitivity (%), specificity (%), and *F*1‐measure (%) scores. Given the basic statistics of true positive (TP) represents the number of risk‐taking managers detected correctly, true negative (TN) measures the number of risk‐averse managers predicted correctly, false negative (FN) represents the number of risk‐taking managers detected as risk‐averse managers, and false positive (FP) represents the number of risk‐averse managers predicted as risk‐taking managers (Farhoumandi et al., 2021). Therefore, the evaluation metrics can be calculated as follows:

(1) $$\mathrm{Accuracy}\;=\;\frac{\mathrm{TP}+\mathrm{TN}}{\mathrm{TP}+\mathrm{TN}+\mathrm{FP}+\mathrm{FN}\;}$$

(2) $$\mathrm{Sensitivity}\;=\frac{\mathrm{TP}\;}{\mathrm{TP}+\mathrm{FN}}$$

(3) $$\mathrm{Specifcity}\;=\frac{\mathrm{TN}\;}{\mathrm{TN}+\mathrm{FP}}$$

(4) $$\mathrm{Precision}\;=\frac{\mathrm{TP}\;}{\mathrm{TP}+\mathrm{FP}}$$

(5) $$F1-\mathrm{measure}\;=\;2\times \;\frac{\mathrm{precision}\times \mathrm{sensitivity}}{\mathrm{precision}+\mathrm{sensitivity}}=\frac{2\mathrm{TP}}{2\mathrm{TP}+\mathrm{FP}+\mathrm{FN}}$$

Accuracy represents the overall effectiveness of the classifier. Moreover, the question of how effectively the classifier identifies risk‐taking and risk‐averse groups is expressed by sensitivity and specificity, respectively. A single metric's combination of precision and sensitivity is shown as the *F*1‐measure.

#### 2.4.3 Statistical methods

For statistical significance of the performance of classifier between size of analysis windows, we used the *t*‐test with Bonferroni's multiple comparison test. *p* < .01 was considered statistically significant.

## 3 RESULTS

**FIGURE 3:**
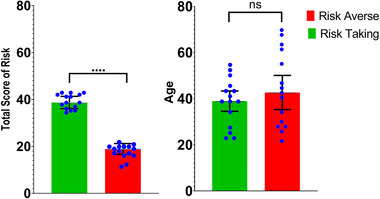
Descriptive statistics of behavioral data and the results of the *t*‐test as the statistical analysis. “****” Indicates *p* < .00001.

In this study, 30 male managers were included in the analysis. The results of the risk‐tolerance scale divide the participants into two groups, risk‐taking and risk‐averse each group consists of 15 participants. The scores can vary from 13 to 47, which indicates a greater willingness to take risks. The scores in the range of 13–21 and 28–47 indicate risk‐averse and risk‐taking managers, respectively (Jann et al., 2010). The 13‐item risk‐tolerance scale questionnaire also is consistent with the self‐evaluation of the managers. The demographic information of the participants, the values of the risk‐tolerance scale, and the results of the *t*‐test as the statistical analysis are shown in Figure 3. There was not a significant difference in age of participants between risk‐taking (M = 38.93, SD = 4.4) and risk‐averse groups (M = 42.66, SD = 6.40); *t*(28) = −1.60, *p* = .124. Also Figure 3 shows the significant difference in the total score of risk based on tolerance scale for risk‐taking group (M = 38.73, SD = 2.61) and risk‐averse group (M = 18.93, SD = 2.30); *t*(28) = 16.65, *p* = .001.

**FIGURE 4:**
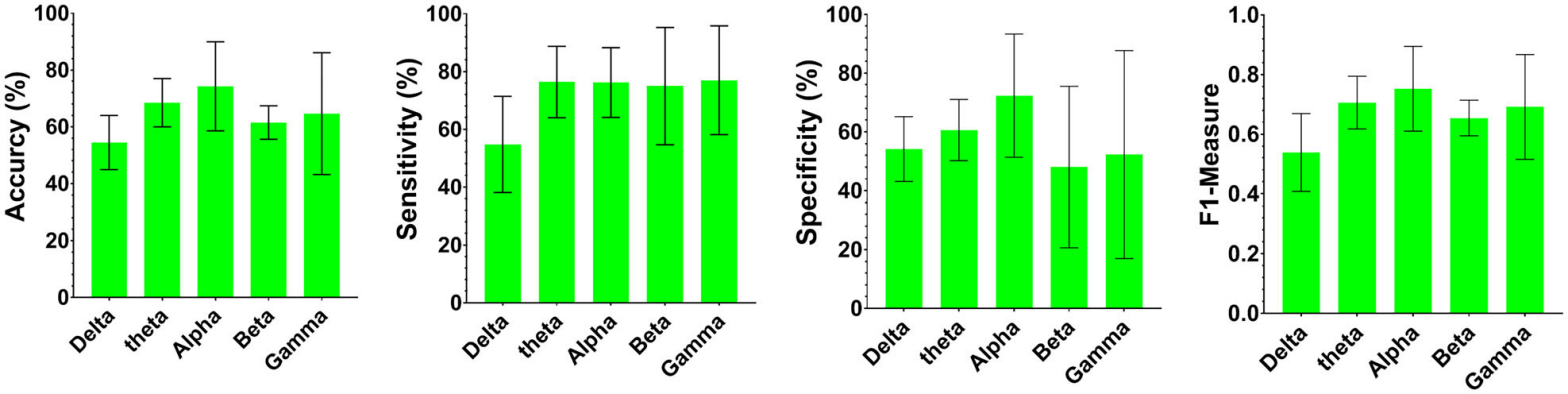
Classification performance for each resting‐state signal frequency band (delta, theta, alpha, beta, and gamma) in 10 s time window.

Based on resting‐state EEG signals, managers were classified into two risk‐taking and risk‐averse groups using different extracted features, the feature selection method, and the SVM classifier. Statistical features are extracted in the time‐frequency domain of brain signals. The two‐step feature selection method was applied to the extracted features to remove bad features and select good ones. The evaluation of classifier performance, which includes testing and training, is based on the fivefold cross‐validation. Figure 4 shows the classification performance for each resting‐state signal frequency band. Notably, the SVM classifier with RBF kernel in the alpha band performs better than the other frequency bands, whereas the results for the theta band are also very close to the alpha band values. The classifier could separate the two groups of managers with 74.42% accuracy, 76.16% sensitivity, 72.32% specificity, and 75% *F*1‐measure using the features extracted from the alpha band in 10 s analysis window size.

**FIGURE 5:**
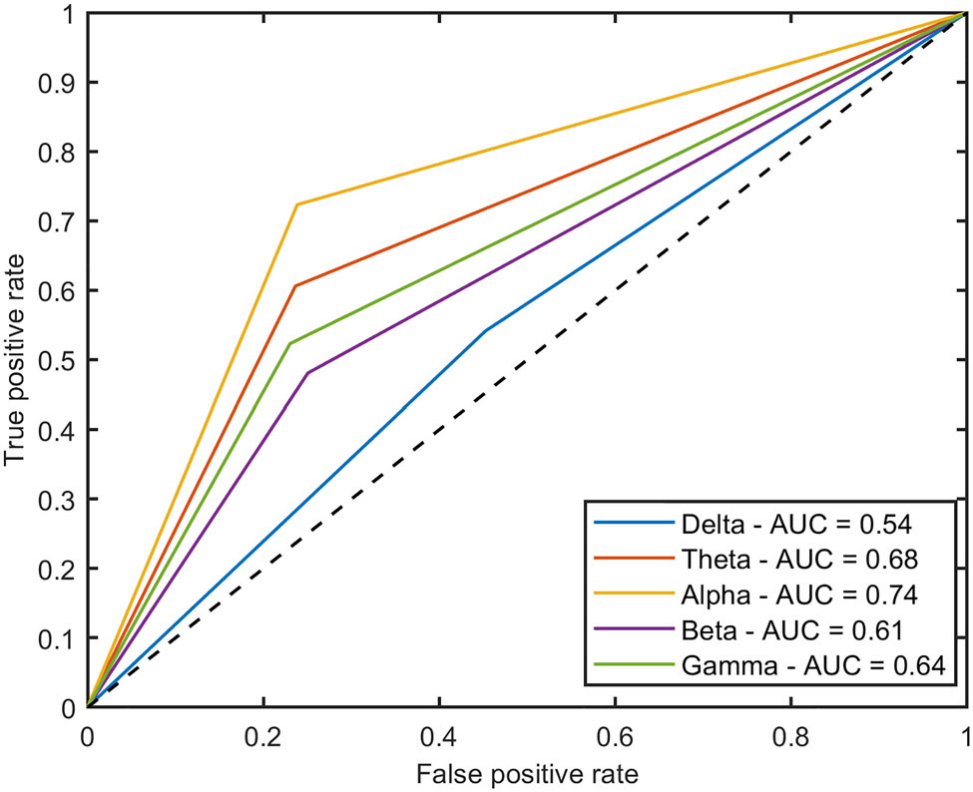
The classification receiver operating characteristic (ROC) curve for each resting‐state signal frequency band (delta, theta, alpha, beta, and gamma) in 10 s time window.

Receiver operating characteristic is shown in Figure 5. As Figure 5 illustrates, the area under curve (AUC) is the measure of the ability of a binary classifier to distinguish between risk‐taking and risk‐averse managers. The AUC for classification performance in alpha frequency band is equal to 0.74. Moreover, the AUC value for gamma, beta, theta, and delta frequency bands is equal to 0.64, 0.61, 0.68, and 0.54, respectively.

**FIGURE 6:**
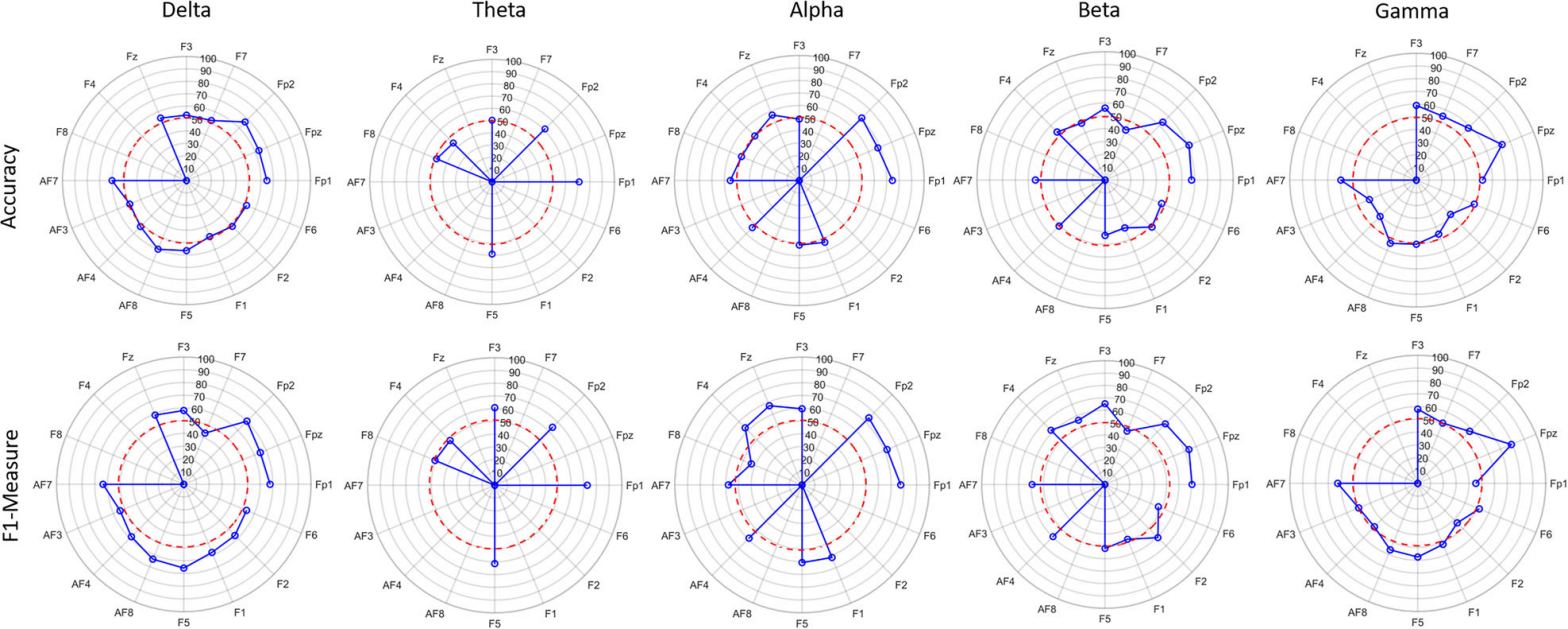
The accuracy and *F*1‐measure of the support vector machine (SVM) classification model using the features extracted from each channel in five frequency bands with 10 s analysis time window.

To investigate the effect of each of the 16 channels of the frontal region, in this study, we separately trained and tested the SVM classification model using the features extracted from each channel with 10 s analysis window size. The accuracy measure and *F*1‐measure resulting from this evaluation are shown in Figure 6.

**FIGURE 7:**
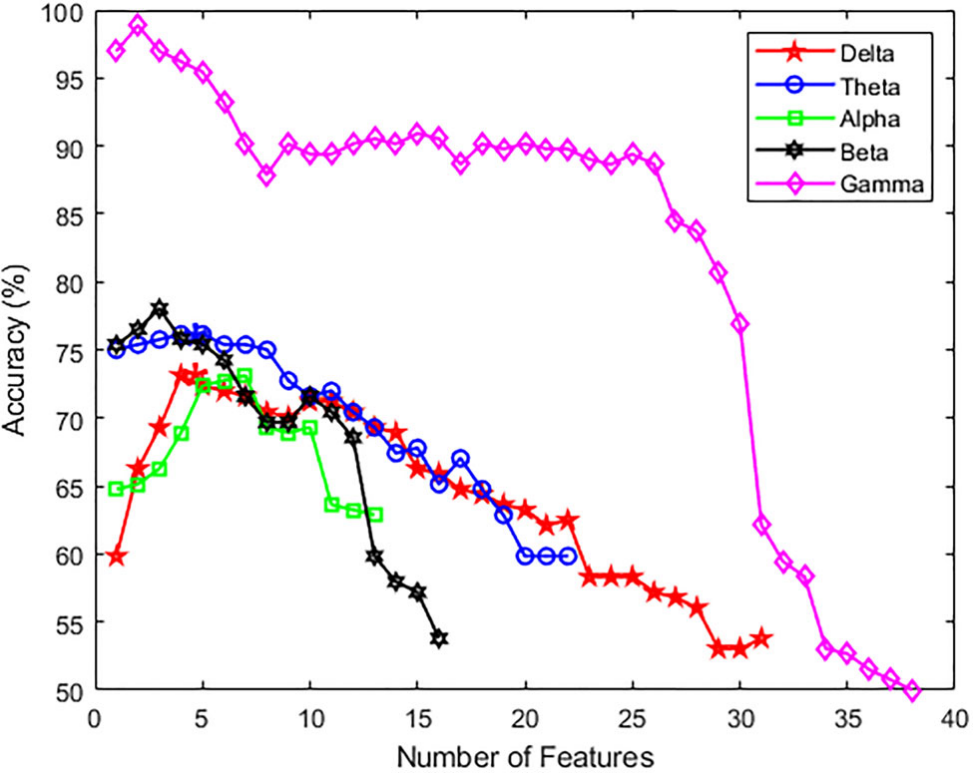
Accuracy and number of features (analysis time window = 10 s) during training and feature selection phases.

To gain insight into how the number of selected features in each frequency band could potentially impact the accuracy of the classification model, we plotted a diagram of accuracy against the number of features (Figure 7) during training and feature selection phases. Our results indicate that as the number of features is increased in all frequency bands, the accuracy of the classification model decreased. This suggests that the proposed method works with a higher accuracy when fewer features are used, which in turn reduces the complexity of the model.

In addition, we have investigated the effect of signal length on classification accuracy. Shortening the time of EEG signals could potentially improve classification accuracy, as it may reduce the amount of noise in the data. However, it is important to balance this with the risk of losing important information that could be useful for classification. It may be useful to see how different signal lengths affect classification performance. Therefore, we have analyzed the classification process with two shorter time windows (2 and 5 s). The results are presented in Figures S1–S4. The statistical test does not show a significant difference in the performance of the classifier with the length of different analysis time windows.

In this study, it should be mentioned the *F*1‐measures were calculated using risk‐taking managers as positive examples. However, if we were to define risk‐averse managers as positive examples instead, the *F*1‐measures would be computed differently. In this scenario, we would consider the risk‐averse managers as positive examples and the risk‐taking managers as negative examples.

To clarify, using this alternate definition, a TP would represent the number of risk‐averse managers correctly detected, a TN would measure the number of risk‐taking managers correctly predicted, a FN would represent risk‐averse managers incorrectly detected as risk‐taking managers, and a FP would represent risk‐taking managers incorrectly predicted as risk‐averse managers. These definitions would yield a different set of values for precision, recall, and the *F*1‐measure compared to when risk‐taking managers are considered positive examples.

It is important to note that the choice of positive examples can significantly impact the performance metrics and should be carefully considered based on the specific goals and context of the analysis. Furthermore, we performed analyses for both assumptions in different frequency bands (Figure S5). As shown, there is no significant difference in the obtained *F*1‐measures across the frequency bands.

## 4 DISCUSSION

This feasibility study aims to assess the effectiveness of using resting‐state EEG signals to identify risk‐taking and risk‐averse managers via ML models. To achieve this goal, statistical features were extracted from the time‐frequency domain of the resting‐state EEG signals. Then, suitable features are selected for classification using a two‐step algorithm. In the first step, a statistical analysis is used to remove bad features. The SFFS algorithm is used to select good features appropriate to the classification in the second step. For the SVM classifier, the best results were obtained in the alpha band with an accuracy of 74.42%, a sensitivity of 76.16%, and a specificity of 72.32% (Figure 4). These results perform better than the SVM classifier values for punishment and reward classifications (58.5% accuracy) in the beta band (Wojcik et al., 2019).

Previous studies applying classic statistical methods to the resting state have suggested a relationship between the risky decision‐making processes and brain signals (Massar et al., 2014; Nakao et al., 2013; Ramsøy et al., 2018; Studer et al., 2013; Yaple et al., 2018). For instance, Studer et al. (2013) found no significant correlation between risk‐taking behavior and overall bilateral prefrontal cortex (PFC) power or PFC asymmetry index in the alpha frequency band. However, these inconsistent findings highlight the need for further investigation. By focusing on the power of alpha frequency in 16 channels (with a significant difference in FP*z*), our study suggests that this frequency band may have a potential role in predicting risky behavior, as indicated by the features calculated based on this power.

Modern automatic methods, such as ML techniques, have been used to classify a variety of diseases and psychological disorders in recent years (Craik et al., 2019; Hosseinifard et al., 2013; Müller et al., 2008; Mumtaz et al., 2017). Although attempts have been made to implement ML models in predicting risky decision‐making in recent years (Si et al., 2020; Wojcik et al., 2019), these methods have not become common. Therefore, using ML models based on EEG signals to evaluate and diagnose risky decision‐making is of great importance. This classification study considers two novel aspects: (1) classification of two groups of (risk‐taking and risk‐averse) managers and (2) classification/prediction at the intersubject (cross‐subject) level.

Although much research has been done on decision‐making and cortical activity, there is still little information about resting neuronal activity in risk‐taking and risk‐averse managers. Despite predicting individual responses in some previous studies (Si et al., 2020; Wojcik et al., 2019), the automatic separation of managers based on resting‐state brain activity has not been studied hitherto, especially at the intersubject level. Si et al. (2020) proposed an ML model for predicting individuals’ responses by extracting distinctive spatial network pattern features from single‐experimental brain networks. The authors presented an LDA‐based model that could use DSNP consistently to achieve higher accuracy than network properties. Seven different classifiers were also used to compare their efficiency in the reward/punishment characteristic cortical activity detection and the punishment and reward classification tasks. The proposed ML models focus only on classifying an individual response, an intra‐subject classification, but this study trained an ML model using selected features that could distinguish risk‐taking and risk‐averse individuals. This issue becomes important when these individuals are managers, which, to our knowledge, have not been studied so far (Wojcik et al., 2019).

Our findings reveal that risk‐taking and risk‐averse managers can be identified using signals recorded from the frontal lobe. These results confirm the previous findings that show the role of the PFC in executive control and maintaining goals in decision‐making and the task of creating fundamental impulses related to self‐interest, which is a consultative process in human decision‐making (Henrich et al., 2001; Huerta & Kaas, 1990; Munakata et al., 2011).

Our findings also suggest that the accuracy of the classification model decreases as the number of features is increased in all frequency bands. These findings have important implications for the practical application of our approach, as it suggests that a simpler model with fewer features may be more effective in identifying risk‐taking and risk‐averse managers using resting‐state EEG signals.

Nevertheless, our proposed methodology suffers from some limitations. In this study, EEG signals were recorded from 30 male managers. In order to avoid the influence of deference gender on decision‐making as well as the small number of female managers, only male participants were used in this study. This has made the ML model unable to learn the unique characteristics of risk‐taking and risk‐aversion. Risk‐taking and risk‐aversion are not limited to managers and all people may be risk‐taking and risk‐averse. However, our study was designed to investigate the neural mechanisms underlying risk aversion in a specific population (i.e., financial decision‐making professionals). Another limitation that we faced in this study was the lack of access to a large number of managers. Only 30 managers have been used in this market. Other limitations related to this study include lack of out‐of‐sample testing. Despite these limitations, our study demonstrates the promising potential of using intelligent systems to select managers based on resting‐state biological signals which avoid potential confounding effects of external stimuli.

## 5 CONCLUSIONS

This study provides compelling evidence for the potential of ML models to identify risk‐taking and risk‐averse managers based on resting‐state EEG signals. It also highlights the importance of exploring the relationship between intrinsic neural properties and risky behavior, as the observed changes in brain signals during this paradigm are primarily driven by internal processes rather than external stimuli. This research has significant implications for future studies in the field of managerial decision‐making, particularly in the development of more sophisticated and accurate predictive models. This feasibility study highlights the possible contribution of these techniques in assigning individuals to highly demanding situations. To achive this goal, future studies’ design should consider groups including moderate levels of risky behavior and an independent sample of participants to assess the generalizability of findings. Progress in the research area might also benefit from using cognitive tasks including questions with real incentives to elicit risk preferences. We hope current study may provide valuable insights into the neural mechanisms underlying risk aversion and can guide future research in this area. The approach could be helpful, especially in the case of leading managers’ decisions that could reasonably impact a country's economic and political conditions.

## AUTHOR CONTRIBUTIONS

**Reza Eyvazpour**: Data curation; software; writing—original draft preparation. **Farhad Farkhondeh Tale Navi**: Writing—reviewing and editing; validation. **Elmira Shakeri**: Visualization; investigation. **Behzad Nikzad**: Statistical analysis. **Soomaayeh Heysieattalab**: Conceptualization; methodology; writing—reviewing and editing; supervision

## CONFLICT OF INTEREST STATEMENT

The authors have no conflicts of interest.

## FUNDING INFORMATION

This research received no specific grant from any funding agency in the public, commercial, or not‐for‐profit sectors.

### PEER REVIEW

The peer review history for this article is available at https://publons.com/publon/10.1002/brb3.3139.

## Supporting information

Figure S1 The classification performance for each resting‐state signal frequency band (delta, theta, alpha, beta, and gamma) and different analysis windows size (ws = 2, 5, and 10 s).
Figure S2 Feature selection shows the number of selected features in each frequency band that has the highest accuracy: (a) analysis windows = 2 s, (b) analysis windows = 5 s during training and feature selection phases.
Figure S3 The classification ROC curve in each resting‐state signal frequency band (delta, theta, alpha, beta, and gamma): (a) analysis windows = 2 s, (b) analysis windows = 5 s.
Figure S4 The accuracy measure and *F*1‐measure were obtained from the SVM classification model using the features extracted from each channel in five frequency bands: (a) analysis windows = 2 s, (b) time windows = 5 s.
Figure S5 *F*1‐measures computed under two alternative assumptions across the frequency bands.

Supporting Information

Supporting Information

Supporting Information

Supporting Information

Supporting Information

Supporting Information

Supporting Information

Supporting Information

## Data Availability

The datasets generated during and/or analyzed during the current study are available from the corresponding author upon reasonable request.

## References

[brb33139-bib-0001] Al‐Nafjan, A. , Hosny, M. , Al‐Ohali, Y. , & Al‐Wabil, A. (2017). Review and classification of emotion recognition based on EEG brain‐computer interface system research: A systematic review. Applied Sciences, 7(12), 1239. 10.3390/app7121239

[brb33139-bib-0002] Anjum, M. F. , Dasgupta, S. , Mudumbai, R. , Singh, A. , Cavanagh, J. F. , & Narayanan, N. S. (2020). Linear predictive coding distinguishes spectral EEG features of Parkinson's disease. Parkinsonism & Related Disorders, 79, 79–85. 32891924 10.1016/j.parkreldis.2020.08.001 PMC7900258

[brb33139-bib-0003] Bajaj, N. (2021). Wavelets for EEG analysis. In Wavelet theory. IntechOpen. 10.5772/intechopen.94398

[brb33139-bib-0004] Bartra, O. , Mcguire, J. T. , & Kable, J. W. (2013). The valuation system: A coordinate‐based meta‐analysis of BOLD fMRI experiments examining neural correlates of subjective value. Neuroimage, 76, 412–427. 10.1016/j.neuroimage.2013.02.063 23507394 PMC3756836

[brb33139-bib-0005] Craik, A. , He, Y. , & Contreras‐Vidal, J. L. (2019). Deep learning for electroencephalogram (EEG) classification tasks: A review. Journal of Neural Engineering, 16(3), 031001. 10.1088/1741-2552/ab0ab5 30808014

[brb33139-bib-0006] Delorme, A. , & Makeig, S. (2004). EEGLAB: An open‐source toolbox for analysis of single‐trial EEG dynamics including independent component analysis. Journal of Neuroscience Methods, 134(1), 9–21. 10.1016/j.jneumeth.2003.10.009 15102499

[brb33139-bib-0007] Farhoumandi, N. , Mollaey, S. , Heysieattalab, S. , Zarean, M. , & Eyvazpour, R. (2021). Facial emotion recognition predicts alexithymia using machine learning. Computational Intelligence and Neuroscience, 2021, 2053795. 34621306 10.1155/2021/2053795 PMC8492233

[brb33139-bib-0008] Gianotti, L. R. R. , Knoch, D. , Faber, P. L. , Lehmann, D. , Pascual‐Marqui, R. D. , Diezi, C. , Schoch, C. , Eisenegger, C. , & Fehr, E. (2009). Tonic activity level in the right prefrontal cortex predicts individuals' risk taking. Psychological Science, 20(1), 33–38. 10.1111/j.1467-9280.2008.02260.x 19152538

[brb33139-bib-0009] Grable, J. (1999). Financial risk tolerance revisited: The development of a risk assessment instrument. Financial Services Review, 8(3), 163–181. 10.1016/S1057-0810(99)00041-4

[brb33139-bib-0010] Grable, J. , & Lytton, R. (2003). The development of a risk assessment instrument: A follow‐up study. Financial Services Review, 12(3), 257. http://search.ebscohost.com/login.aspx?direct=true&profile=ehost&scope=site&authtype=crawler&jrnl=10570810&AN=11356299&h=bW2WU6tJ2FNJuXwi1O3NXP1l27L%2FQ1hrJzxnkEuPnUtOA4lmtTSanW3aLUcaYebLbhElJ%2BKgL21khaaUGZ9Fwg%3D%3D&crl=c

[brb33139-bib-0011] Henrich, J. , Boyd, R. , Bowles, S. , Camerer, C. , Fehr, E. , Gintis, H. , & Mcelreath, R. (2001). In search of Homo economicus: Behavioral experiments in 15 small‐scale societies. American Economic Review, 91(2), 73–78. 10.1257/aer.91.2.73

[brb33139-bib-0012] Hosseinifard, B. , Moradi, M. H. , & Rostami, R. (2013). Classifying depression patients and normal subjects using machine learning techniques and nonlinear features from EEG signal. Computer Methods and Programs in Biomedicine, 109(3), 339–345. 10.1016/j.cmpb.2012.10.008 23122719

[brb33139-bib-0013] Huerta, M. F. , & Kaas, J. H. (1990). Supplementary eye field as defined by intracortical microstimulation: Connections in macaques. Journal of Comparative Neurology, 293(2), 299–330. 10.1002/cne.902930211 19189718

[brb33139-bib-0014] Ieracitano, C. , Mammone, N. , Hussain, A. , & Morabito, F. C. (2020). A novel multi‐modal machine learning based approach for automatic classification of EEG recordings in dementia. Neural Networks, 123, 176–190. 10.1016/j.neunet.2019.12.006 31884180

[brb33139-bib-0015] Ivaskevych, D. (2019). Parietal EEG theta/beta ratio as an electrophysiological marker for extraversion‐related differences. Psychology and Behavioral Science International Journal, 11(3), 555814. 10.19080/PBSIJ.2019.11.555814

[brb33139-bib-0016] Jacob, J. E. , Nair, G. K. , Iype, T. , & Cherian, A. (2018). Diagnosis of encephalopathy based on energies of EEG subbands using discrete wavelet transform and support vector machine. Neurology Research International, 2018, 1–9. 10.1155/2018/1613456 PMC6051006 30057813

[brb33139-bib-0017] Jann, K. , Koenig, T. , Dierks, T. , Boesch, C. , & Federspiel, A. (2010). Association of individual resting state EEG alpha frequency and cerebral blood flow. Neuroimage, 51(1), 365–372. 10.1016/j.neuroimage.2010.02.024 20156573

[brb33139-bib-0018] Krain, A. L. , Wilson, A. M. , Arbuckle, R. , Castellanos, F. X. , & Milham, M. P. (2006). Distinct neural mechanisms of risk and ambiguity: A meta‐analysis of decision‐making. Neuroimage, 32(1), 477–484. 10.1016/j.neuroimage.2006.02.047 16632383

[brb33139-bib-0019] Lee, J. Y. , Park, S. M. , Kim, Y. J. , Kim, D. J. , Choi, S.‐W. , Kwon, J. S. , & Choi, J.‐S. (2017). Resting‐state EEG activity related to impulsivity in gambling disorder. Journal of Behavioral Addictions, 6(3), 387–395. 10.1556/2006.6.2017.055 28856896 PMC5700729

[brb33139-bib-0020] Li, N. , Ma, N. , Liu, Y. , He, X.‐S. , Sun, D.‐L. , Fu, X.‐M. , Zhang, X. , Han, S. , & Zhang, D.‐R. (2013). Resting‐state functional connectivity predicts impulsivity in economic decision‐making. Journal of Neuroscience, 33(11), 4886–4895. 10.1523/JNEUROSCI.1342-12.2013 23486959 PMC6618998

[brb33139-bib-0021] Li, Z. , Zhang, L. , Zhang, F. , Gu, R. , Peng, W. , & Hu, L. (2020). Demystifying signal processing techniques to extract resting‐state EEG features for psychologists. Brain Science Advances, 6(3), 189–209. 10.26599/BSA.2020.9050019

[brb33139-bib-0022] Lin, X. , Featherman, M. , Brooks, S. L. , & Hajli, N. (2019). Exploring gender differences in online consumer purchase decision making: An online product presentation perspective. Information Systems Frontiers, 21(5), 1187–1201. 10.1007/s10796-018-9831-1

[brb33139-bib-0023] Ludwig, K. A. , Miriani, R. M. , Langhals, N. B. , Joseph, M. D. , Anderson, D. J. , & Kipke, D. R. (2009). Using a common average reference to improve cortical neuron recordings from microelectrode arrays. Journal of Neurophysiology, 101(3), 1679–1689. 10.1152/jn.90989.2008 19109453 PMC2666412

[brb33139-bib-0024] Mahaldar, O. , & Aditya, S. (2017). Gender differences in brain activity during exposure to emotional film clips: An EEG study. Cognition, Brain, Behavior, 21(1), 29–53. 10.24193/cbb.2017.21.03

[brb33139-bib-0025] Maitín, A. M. , García‐Tejedor, A. J. , & Muñoz, J. P. R. (2020). Machine learning approaches for detecting Parkinson's disease from EEG analysis: A systematic review. Applied Sciences, 10(23), 8662. 10.3390/app10238662

[brb33139-bib-0026] Massar, S. A. A. , Kenemans, J. L. , & Schutter, D. J. L. G. (2014). Resting‐state EEG theta activity and risk learning: Sensitivity to reward or punishment? International Journal of Psychophysiology, 91(3), 172–177. 10.1016/j.ijpsycho.2013.10.013 24184042

[brb33139-bib-0027] Mehta, R. (2020). Gender‐based differences in consumer decision‐making styles: Implications for marketers. Decision, 47(3), 319–329. 10.1007/s40622-020-00252-8

[brb33139-bib-0028] Müller, K.‐R. , Tangermann, M. , Dornhege, G. , Krauledat, M. , Curio, G. , & Blankertz, B. (2008). Machine learning for real‐time single‐trial EEG‐analysis: From brain‐computer interfacing to mental state monitoring. Journal of Neuroscience Methods, 167(1), 82–90. 10.1016/j.jneumeth.2007.09.022 18031824

[brb33139-bib-0029] Mumtaz, W. , Vuong, P. L. , Xia, L. , Malik, A. S. , & Rashid, R. B. A. (2017). An EEG‐based machine learning method to screen alcohol use disorder. Cognitive Neurodynamics, 11(2), 161–171. 10.1007/s11571-016-9416-y 28348647 PMC5350086

[brb33139-bib-0030] Munakata, Y. , Herd, S. A. , Chatham, C. H. , Depue, B. E. , Banich, M. T. , & O'reilly, R. C. (2011). A unified framework for inhibitory control. Trends in Cognitive Sciences, 15(10), 453–459. 10.1016/j.tics.2011.07.011 21889391 PMC3189388

[brb33139-bib-0031] Nakao, T. , Bai, Y. , Nashiwa, H. , & Northoff, G. (2013). Resting‐state EEG power predicts conflict‐related brain activity in internally guided but not in externally guided decision‐making. Neuroimage, 66, 9–21. 10.1016/j.neuroimage.2012.10.034 23103687

[brb33139-bib-0032] Noor, N. S. E. M. , & Ibrahim, H. (2020). Machine learning algorithms and quantitative electroencephalography predictors for outcome prediction in traumatic brain injury: A systematic review. IEEE Access, 8, 102075–102092. 10.1109/ACCESS.2020.2998934

[brb33139-bib-0033] Opris, I. , Ionescu, S. C. , Lebedev, M. A. , Boy, F. , Lewinski, P. , & Ballerini, L. (2020). Editorial: Application of neural technology to neuro‐management and neuro‐marketing. Frontiers in Neuroscience, 14, 53. 10.3389/fnins.2020.00053 32116504 PMC7034133

[brb33139-bib-0034] Pornpattananangkul, N. , Grogans, S. , Yu, R. , & Nusslock, R. (2019). Single‐trial EEG dissociates motivation and conflict processes during decision‐making under risk. Neuroimage, 188, 483–501. 10.1016/j.neuroimage.2018.12.029 30557662 PMC6401252

[brb33139-bib-0035] Poudel, R. , Riedel, M. C. , Salo, T. , Flannery, J. S. , Hill‐Bowen, L. D. , Eickhoff, S. B. , Laird, A. R. , & Sutherland, M. T. (2020). Common and distinct brain activity associated with risky and ambiguous decision‐making. Drug and Alcohol Dependence, 209, 107884. 10.1016/j.drugalcdep.2020.107884 32078973 PMC7127964

[brb33139-bib-0036] Pudil, P. , Novovičová, J. , & Kittler, J. (1994). Floating search methods in feature selection. Pattern Recognition Letters, 15(11), 1119–1125. 10.1016/0167-8655(94)90127-9

[brb33139-bib-0037] Ramsøy, T. Z. , Skov, M. , Christensen, M. K. , & Stahlhut, C. (2018). Frontal brain asymmetry and willingness to pay. Frontiers in Neuroscience, 12, 138. 10.3389/fnins.2018.00138 29662432 PMC5890093

[brb33139-bib-0038] Rasheed, K. , Qayyum, A. , Qadir, J. , Sivathamboo, S. , Kwan, P. , Kuhlmann, L. , O'brien, T. , & Razi, A. (2020). Machine learning for predicting epileptic seizures using EEG signals: A review. IEEE Reviews in Biomedical Engineering, 14, 139–155. 10.1109/RBME.2020.3008792 32746369

[brb33139-bib-0039] Roy, Y. , Banville, H. , Albuquerque, I. , Gramfort, A. , Falk, T. H. , & Faubert, J. (2019). Deep learning‐based electroencephalography analysis: A systematic review. Journal of Neural Engineering, 16(5), 051001. 10.1088/1741-2552/ab260c 31151119

[brb33139-bib-0041] Schutter, D. J. L. G. , & Van Honk, J. (2005). Electrophysiological ratio markers for the balance between reward and punishment. Cognitive Brain Research, 24(3), 685–690. 10.1016/j.cogbrainres.2005.04.002 15878265

[brb33139-bib-0042] Shanir, P. P. M. , Khan, K. A. , Khan, Y. U. , Farooq, O. , & Adeli, H. (2018). Automatic seizure detection based on morphological features using one‐dimensional local binary pattern on long‐term EEG. Clinical EEG and Neuroscience, 49(5), 351–362. 10.1177/1550059417744890 29214865

[brb33139-bib-0043] Si, Y. , Jiang, L. , Tao, Q. , Chen, C. , Li, F. , Jiang, Y. , Zhang, T. , Cao, X. , Wan, F. , Yao, D. , & Xu, P. (2019). Predicting individual decision‐making responses based on the functional connectivity of resting‐state EEG. Journal of Neural Engineering, 16(6), 066025. 10.1088/1741-2552/ab39ce 31394516

[brb33139-bib-0044] Si, Y. , Li, F. , Duan, K. , Tao, Q. , Li, C. , Cao, Z. , Zhang, Y. , Biswal, B. , Li, P. , Yao, D. , & Xu, P. (2020). Predicting individual decision‐making responses based on single‐trial EEG. Neuroimage, 206, 116333. 10.1016/j.neuroimage.2019.116333 31698078

[brb33139-bib-0045] Srivastava, M. , Sharma, G. D. , Srivastava, A. K. , & Kumaran, S. S (2020). What's in the brain for us: A systematic literature review of neuroeconomics and neurofinance. Qualitative Research in Financial Markets, 12(4), 413–435. 10.1108/QRFM-10-2019-0127

[brb33139-bib-0046] Studer, B. , Pedroni, A. , & Rieskamp, J. (2013). Predicting risk‐taking behavior from prefrontal resting‐state activity and personality. PLoS One, 8(10), e76861. 10.1371/journal.pone.0076861 24116176 PMC3792091

[brb33139-bib-0047] Tzimourta, K. D. , Christou, V. , Tzallas, A. T. , Giannakeas, N. , Astrakas, L. G. , Angelidis, P. , Tsalikakis, D. , & Tsipouras, M. G. (2021). Machine learning algorithms and statistical approaches for Alzheimer's disease analysis based on resting‐state EEG recordings: A systematic review. International Journal of Neural Systems, 31(05), 2130002. 10.1142/S0129065721300023 33588710

[brb33139-bib-0048] Viacava, K. R. , Vigo, A. , & Bizarro, L. (2016). Process evaluation of an applied neuroeconomics extension course in consulting and management based on Kirkpatrick's model. Journal of Management Research, 8(4), 22. 10.5296/jmr.v9i1.10374

[brb33139-bib-0049] Wang, Z. , & Mengoni, P. (2022). Seizure classification with selected frequency bands and EEG montages: A natural language processing approach. Brain Informatics, 9(1), 1–31. 10.1186/s40708-022-00159-3 35622175 PMC9142724

[brb33139-bib-0050] Wilson, M. J. , & Vassileva, J. (2018). Decision‐making under risk, but not under ambiguity, predicts pathological gambling in discrete types of abstinent substance users. Frontiers in Psychiatry, 9, 1–10. 10.3389/fpsyt.2018.00239 29922190 PMC5996080

[brb33139-bib-0051] Wojcik, G. M. , Masiak, J. , Kawiak, A. , Kwasniewicz, L. , Schneider, P. , Postepski, F. , & Gajos‐Balinska, A. (2019). Analysis of decision‐making process using methods of quantitative electroencephalography and machine learning tools. Frontiers in Neuroinformatics, 13, 1–16. 10.3389/fninf.2019.00073 31827431 PMC6892351

[brb33139-bib-0052] Yaple, Z. , Martinez‐Saito, M. , Novikov, N. , Altukhov, D. , Shestakova, A. , & Klucharev, V. (2018). Power of feedback‐induced beta oscillations reflect omission of rewards: Evidence from an EEG gambling study. Frontiers in Neuroscience, 12, 1–11. 10.3389/fnins.2018.00776 30425616 PMC6218571

[brb33139-bib-0053] Zhang, R. (2018). Decision‐making mechanism under economic management risk based on event‐related potential neuroimaging technique. NeuroQuantology, 16(3), 75–82. 10.14704/nq.2018.16.3.1202

[brb33139-bib-0054] Zheng, Y. , Yi, W. , Cheng, J. , & Li, Q. (2020). Common and distinct electrophysiological correlates of feedback processing during risky and ambiguous decision‐making. Neuropsychologia, 146(16), 107526. 10.1016/j.neuropsychologia.2020.107526 32535129

