# Supplementary material for: Machine learning‐based classifying of risk‐takers and risk‐aversive individuals using resting‐state EEG data: A pilot feasibility study
Source: Brain Behav. 2023 Jun 27;13(9):e3139. doi: 10.1002/brb3.3139 (PMC10498077; doi:10.1002/brb3.3139)
Supplement: Supplementary file 1 — Figure S1 The classification performance for each resting‐state signal frequency band (delta, theta, alpha, beta, and gamma) and different analysis windows size (ws = 2, 5, and 10 s). Figure S2 Feature selection shows the number of selected features in each frequency band that has the highest accuracy: (a) analysis windows = 2 s, (b) analysis windows = 5 s during training and feature selection phases. Figure S3 The classification ROC curve in each resting‐state signal frequency band (delta, theta, alpha, beta, and gamma): (a) analysis windows = 2 s, (b) analysis windows = 5 s. Figure S4 The accuracy measure and F1‐measure were obtained from the SVM classification model using the features extracted from each channel in five frequency bands: (a) analysis windows = 2 s, (b) time windows = 5 s. Figure S5 F1‐measures computed under two alternative assumptions across the frequency bands. [file BRB3-13-e3139-s002.docx]

**Supplementary material:** Machine Learning-Based Classifying of Risk-takers and Risk-Aversive Individuals Using Resting-State EEG Data: A Feasibility Study

| 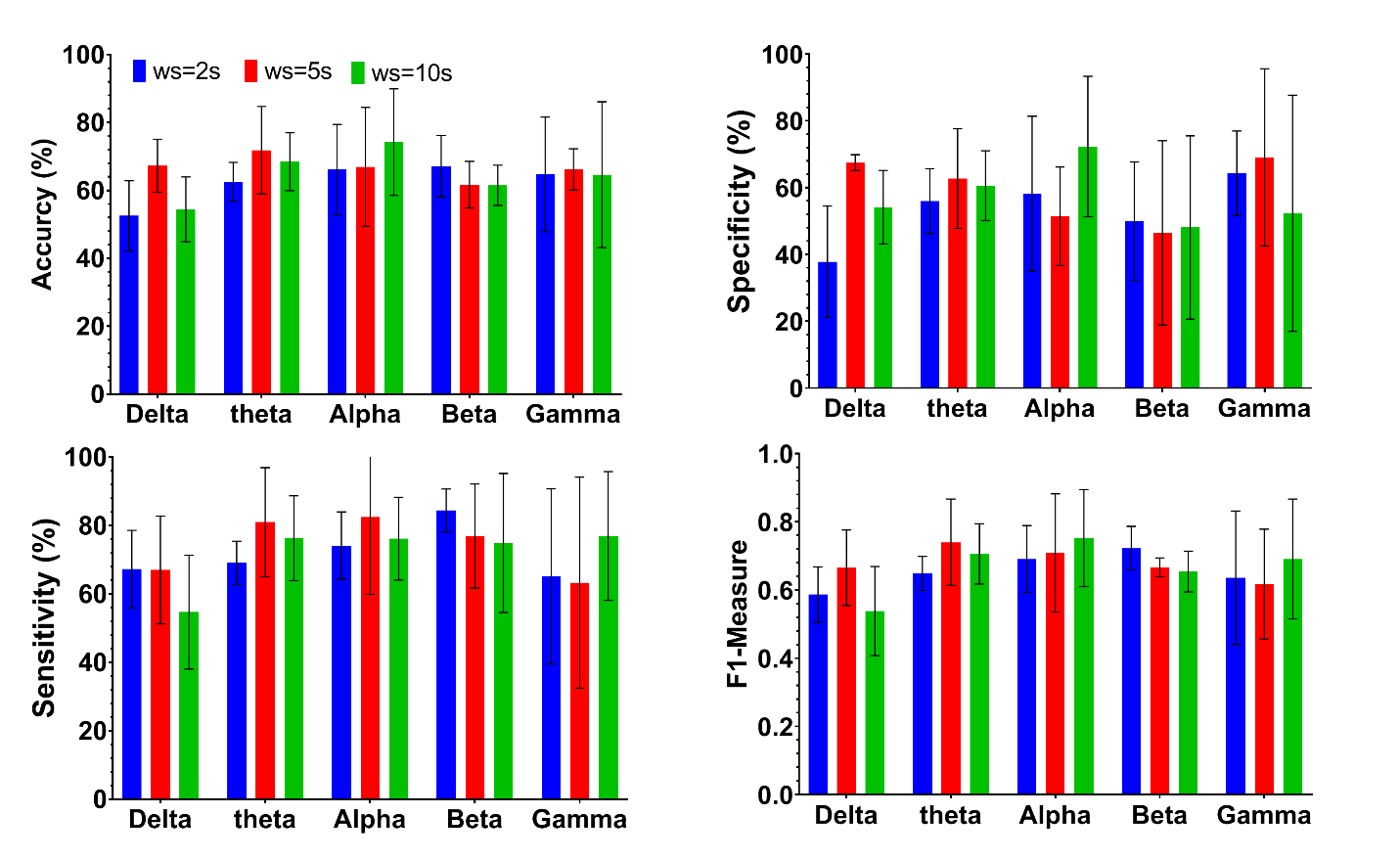 |
| --- |
| **Figure S1.** The classification performance for each resting-state signal frequency band (Delta, Theta, Alpha, Beta, and Gamma) and different analysis windows size (ws = 2, 5, and 10 second) |

a)


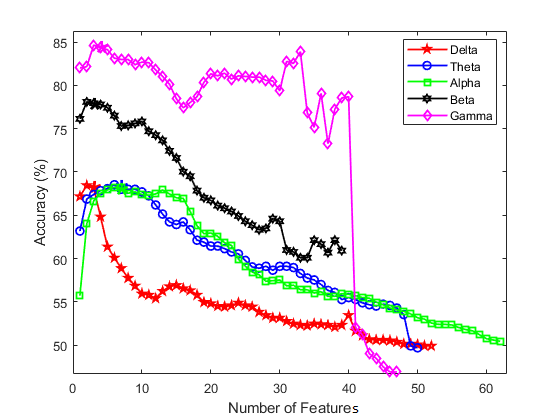


b)


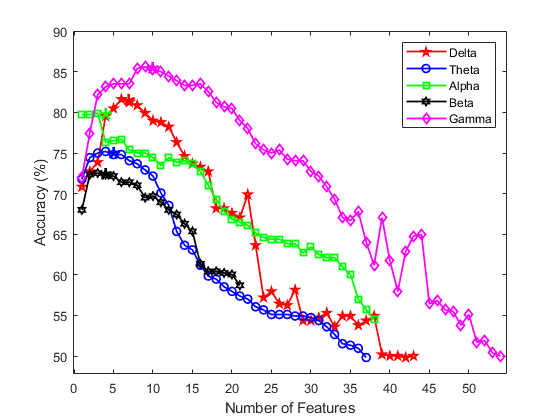


**Figure S2.** Feature selection: shows the number of selected features in each frequency band that have the highest accuracy. a) Analysis windows = 2 s, b) Analysis windows = 5 s) during training and feature selection phases.

| a) | **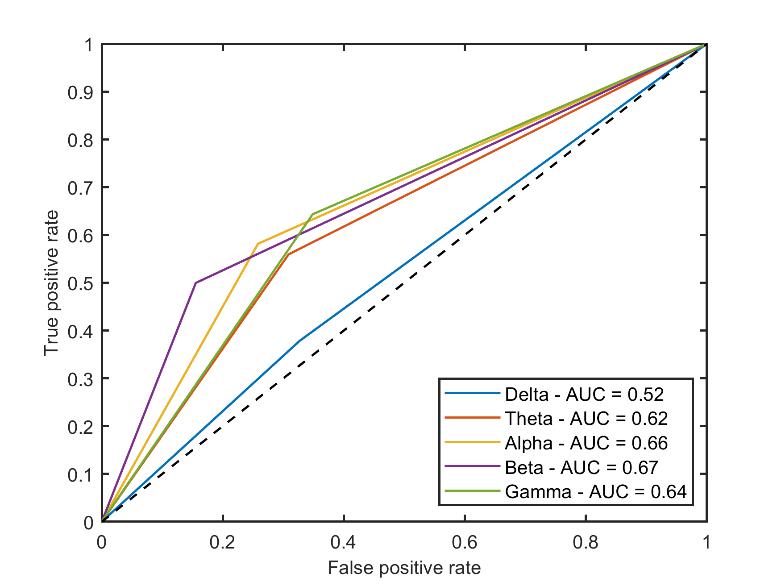** |
| --- | --- |
| b) | **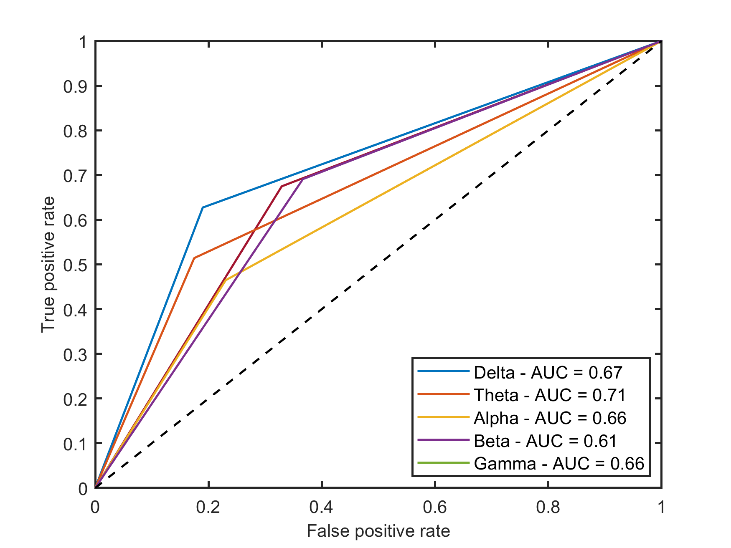** |

**Figure S3.** The classification ROC curve in each resting-state signal frequency band (Delta, Theta, Alpha, Beta, and Gamma). a) Analysis windows= 2s, b) Analysis windows= 5s.

| a) | 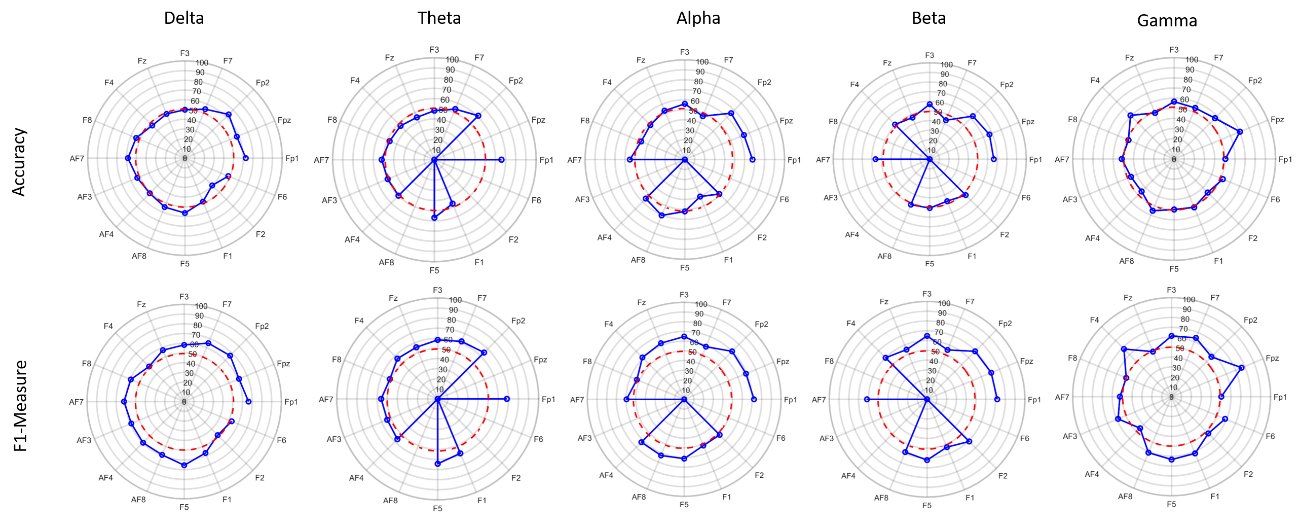 |
| --- | --- |
| b) | 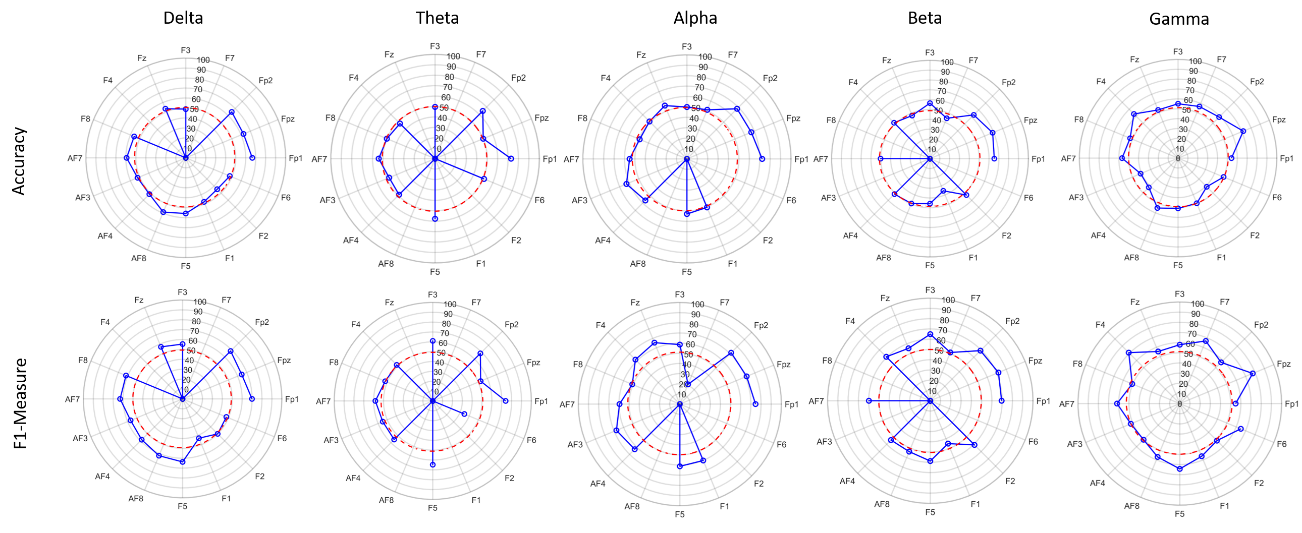 |
| **Figure S4.** The accuracy measure and F1-Measure were obtained from the SVM classification model using the features extracted from each channel in five frequency bands. a) Analysis windows = 2s, b) time windows = 5s. | |

In this study, the F1-measures were calculated using risk-taking managers as positive examples. However, if we were to define risk-averse managers as positive examples instead, the F1-measures would be computed differently. In this scenario, we would consider the risk-averse managers as positive examples and the risk-taking managers as negative examples.

To clarify, using this alternate definition, a true positive (TP) would represent the number of risk-averse managers correctly detected, a true negative (TN) would measure the number of risk-taking managers correctly predicted, a false negative (FN) would represent risk-averse managers incorrectly detected as risk-taking managers, and a false positive (FP) would represent risk-taking managers incorrectly predicted as risk-averse managers. These definitions would yield a different set of values for precision, recall, and the F1-measure compared to when risk-taking managers are considered positive examples.

It is important to note that the choice of positive examples can significantly impact the performance metrics and should be carefully considered based on the specific goals and context of the analysis. Furthermore, we performed analyses for both assumptions in different frequency bands (Figure S.5). As shown, there is no significant difference in the obtained F1-measures across the frequency bands.


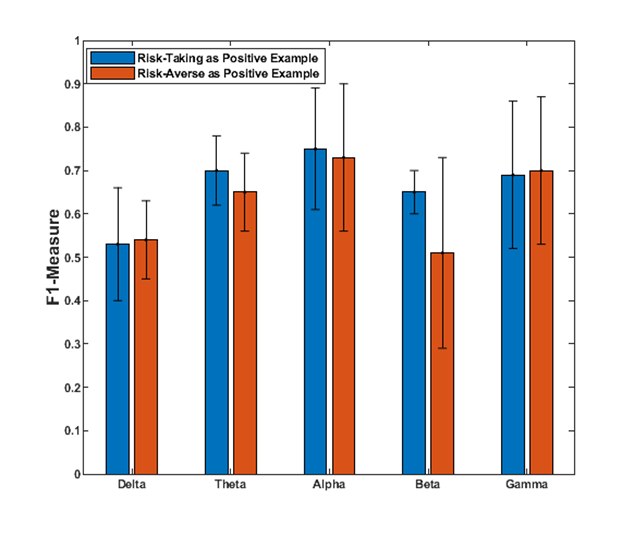


**Figure S.5.** F1-measures computed under two alternative assumptions across the frequency bands.
