# Supplementary figures and images for: Machine learning‐based classifying of risk‐takers and risk‐aversive individuals using resting‐state EEG data: A pilot feasibility study
Source: Brain Behav. 2023 Jun 27;13(9):e3139. doi: 10.1002/brb3.3139 (PMC10498077; doi:10.1002/brb3.3139)

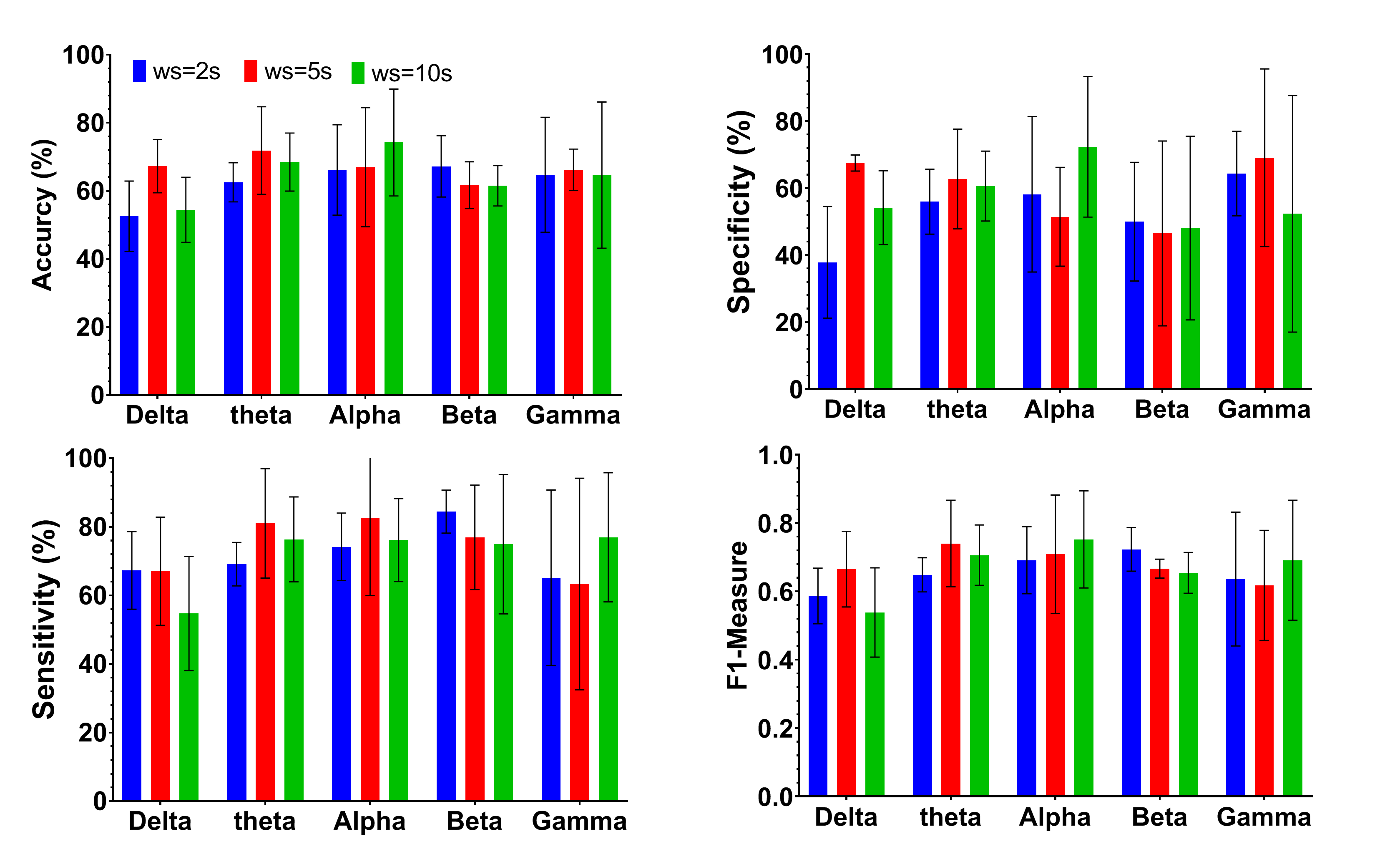

Supplement: Supplementary file 2 — Supporting Information [file BRB3-13-e3139-s009.tif]

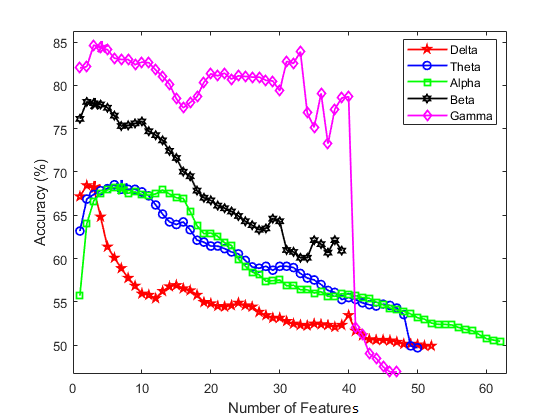

Supplement: Supplementary file 3 — Supporting Information [file BRB3-13-e3139-s001.tif]

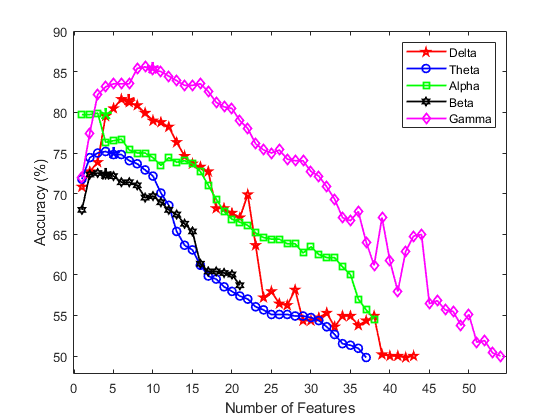

Supplement: Supplementary file 4 — Supporting Information [file BRB3-13-e3139-s006.tif]

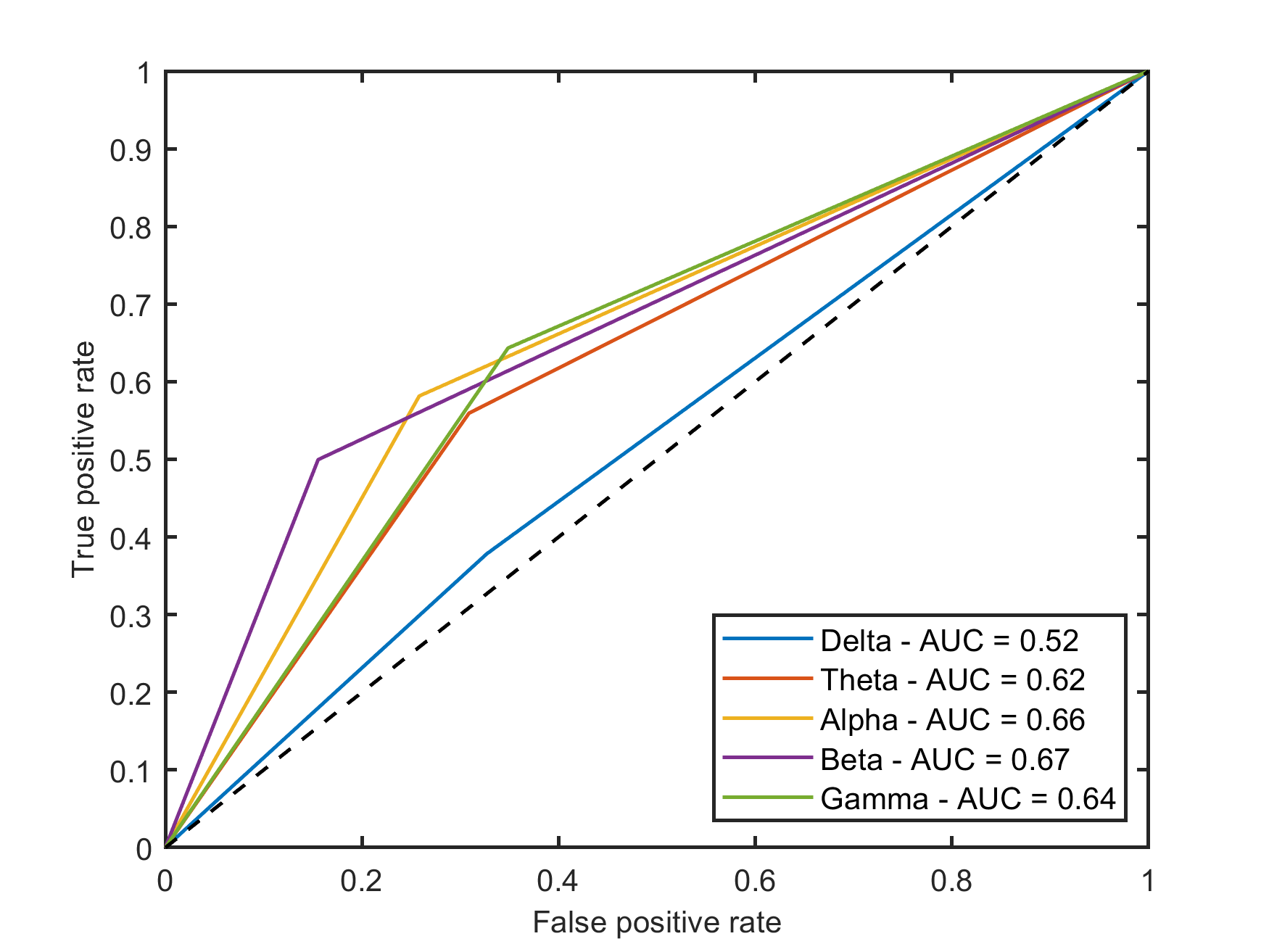

Supplement: Supplementary file 5 — Supporting Information [file BRB3-13-e3139-s007.tif]

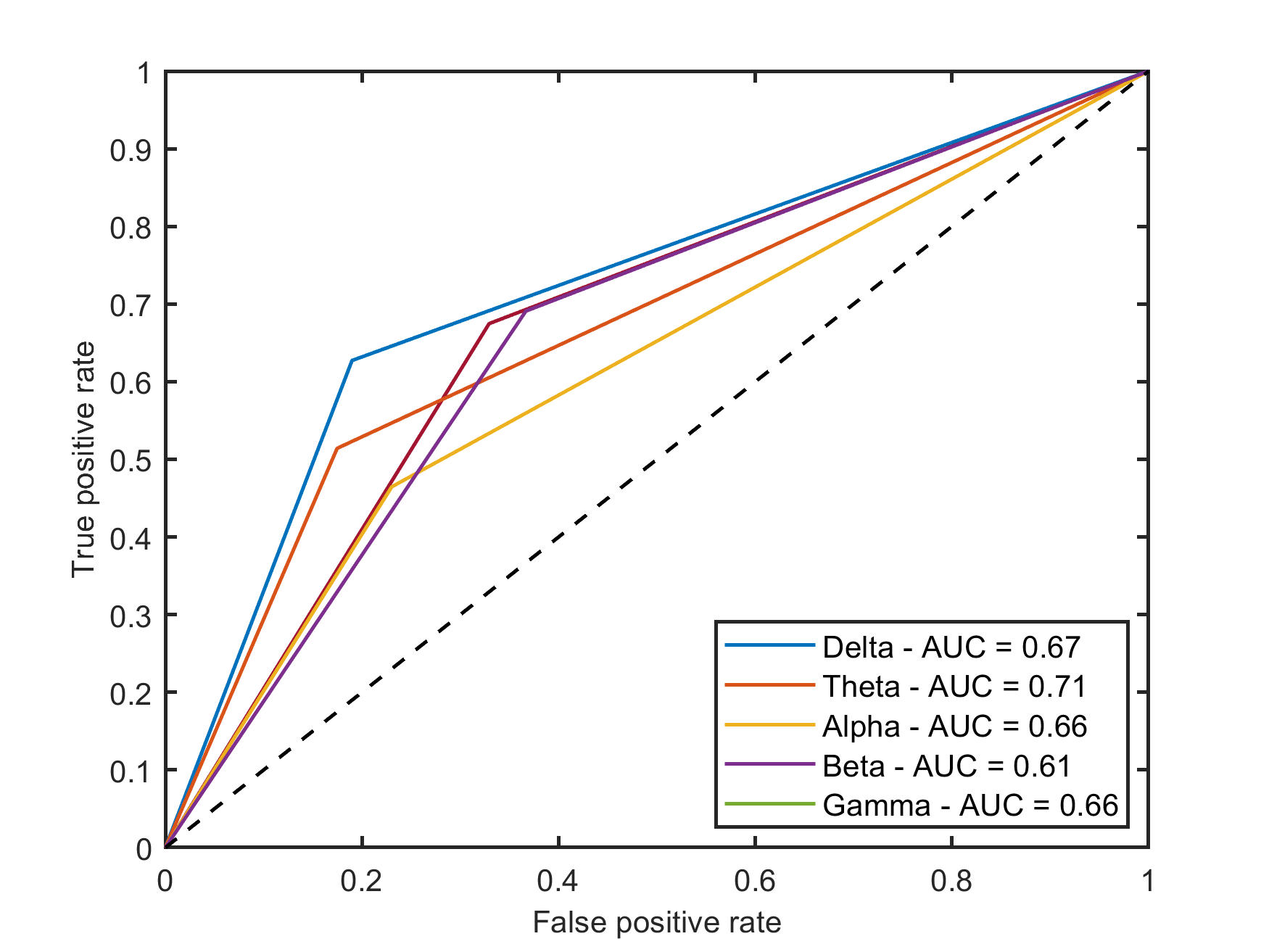

Supplement: Supplementary file 6 — Supporting Information [file BRB3-13-e3139-s005.tif]

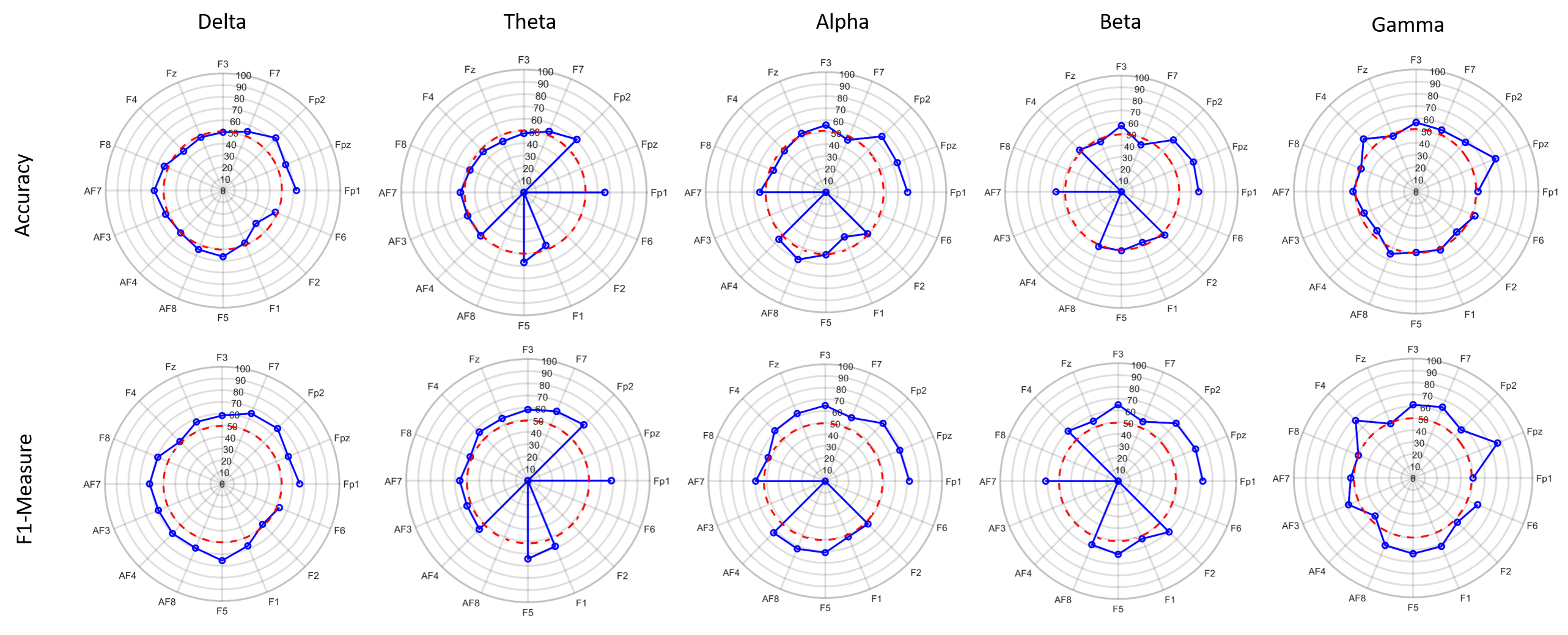

Supplement: Supplementary file 7 — Supporting Information [file BRB3-13-e3139-s004.tif]

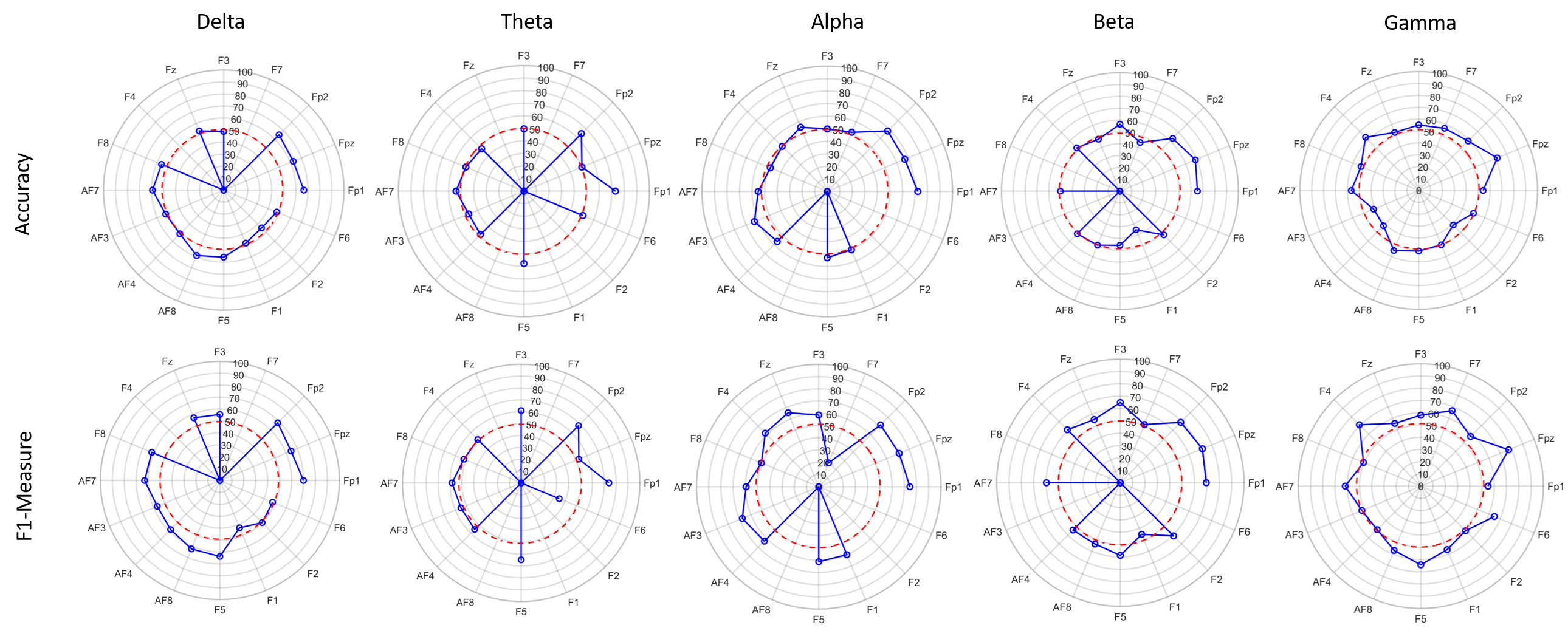

Supplement: Supplementary file 8 — Supporting Information [file BRB3-13-e3139-s003.tif]

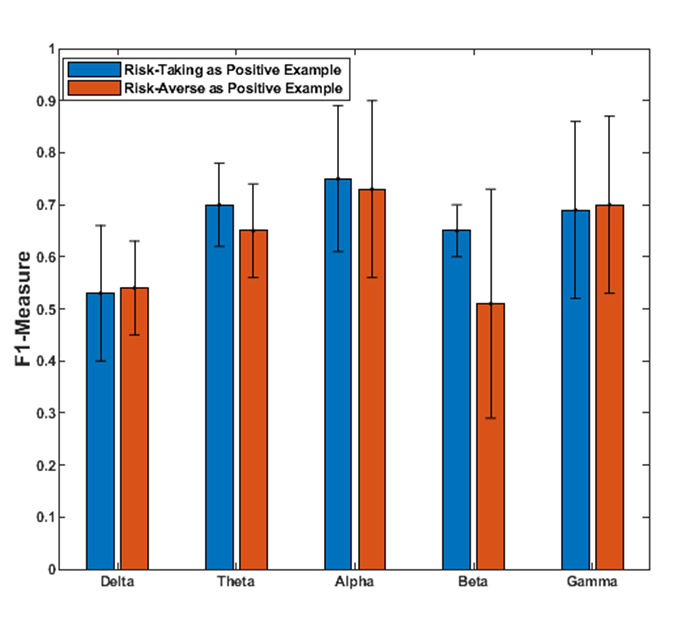

Supplement: Supplementary file 9 — Supporting Information [file BRB3-13-e3139-s008.tif]
